# Supplementary material for: Transcriptional Profiling Reveals Ribosome Biogenesis, Microtubule Dynamics and Expression of Specific lncRNAs to be Part of a Common Response to Cell-Penetrating Peptides
Source: Biomolecules. 2020 Nov 17;10(11):1567. doi: 10.3390/biom10111567 (PMC7698553; doi:10.3390/biom10111567)
Supplement: Supplementary file 1 [file biomolecules-10-01567-s001.zip › proofed manuscript and supplemental data/Supplementary Figures final.docx]

**Figure S1.** (**A**) Heatmap of RNA seq profiles of selected genes. (**B**–**D**) Quantification of selected genes by quantitative RT-PCR.

**Figure S2.** Gene ontology analysis of all Up- and Down-regulated genes found in samples treated for 1- or 4-h.

**Figure S3.** Gene ontology analysis of all Up- and Down-regulated genes found in the intersection between different CPP treated cells.

**Figure S4.** HCS profiling of penetratin treated cells with different cellular markers.
